# Emotion dysregulation in attention-deficit/hyperactivity disorder and borderline personality disorder

**DOI:** 10.1186/s40479-018-0086-8

**Published:** 2018-05-20

**Authors:** Talar R. Moukhtarian, Ruth S. Mintah, Paul Moran, Philip Asherson

**Affiliations:** 1 King’s College London, MRC Social, Genetic and Developmental Psychiatry Centre, Institute of Psychiatry, Psychology and Neuroscience, London, SE5 8AF UK; 2 Centre for Academic Mental Health, Department of Population Health Sciences, Bristol Medical School, University of Bristol, Bristol, BS8 2BN UK

**Keywords:** ADHD, BPD, Emotion dysregulation, Symptomatic overlap

## Abstract

There is ongoing debate on the overlap between Attention-Deficit/Hyperactivity Disorder (ADHD) and Borderline Personality Disorder (BPD), particularly regarding emotion dysregulation (ED). In this paper, we present a narrative review of the available evidence on the association of these two disorders from several standpoints. First, we discuss the unique and shared diagnostic criteria for ADHD and BPD, focusing particularly on ED. We consider the methodology of ecological momentary assessment and discuss why this approach could be an alternative and more accurate way to qualitatively distinguish between ADHD and BPD. We summarise key findings on the genetic and environmental risk factors for ADHD and BPD and the extent to which there are shared or unique aetiological and neurobiological risk factors. Finally, we discuss the clinical relevance of considering both disorders in the assessment of patients presenting with trait-like behavioural syndromes, distinguishing the two conditions and implications for treatment.

## Background

In recent years, a debate has ensued over the nosological distinction between Attention-Deficit/Hyperactivity Disorder (ADHD) and Borderline Personality Disorder (BPD) [1]. Impulsivity, irritability and other symptoms of emotional dysregulation are characteristically seen in both disorders, and the nature of the relationship between ADHD and BPD requires clarification [2]. Key questions that arise include the extent to which: 1) ADHD and BPD co-occur; 2) they reflect distinct disorders or alternative expressions of the same underlying disorder; 3) they share common genetic or environmental risk factors; and 4) one of the disorders give a synergistic effect, reinforcing the other or complicating both [3, 4].

In this review paper, we present a narrative description of the available evidence on the association between ADHD and BPD pertaining specifically to emotional dysregulation (ED). We start by presenting an account of the main diagnostic features of each disorder and outlining the clinical features that are common to BPD and ADHD, then summarising studies that have reported on comorbidity between the two disorders. We then review findings from studies that have measured ED in ADHD and BPD using experience sampling methods, as this provides a precise and ecologically valid way of assessing the phenomenon of ED. Finally, we discuss the extent to which there are shared genetic and environmental risks, and shared neurobiology, for the two disorders, before considering implications of these findings for treatment.

### Attention-deficit/hyperactivity disorder

ADHD is a common neurodevelopmental disorder emerging in childhood or early adolescence, characterised by a pervasive pattern of developmentally inappropriate levels of inattention and/or hyperactivity-impulsivity that lead to clinically significant functional and psychosocial impairments [5]. The disorder affects around 5% of children [6]. Longitudinal follow-up studies of children with ADHD show that symptoms of ADHD commonly persist into adulthood, with around two-thirds of cases meeting either full or sub-threshold criteria in adulthood [7]. The prevalence of adult ADHD in epidemiological surveys is estimated at around 2.5–4% [8–10]. Although ADHD is recognised as a predominantly male disorder in childhood (clinic-referred children are more likely to be male), in adult samples the gender difference is less pronounced [11].

Apart from the main symptoms used to classify ADHD, ED is considered to be an associated feature supporting the diagnosis of ADHD [5, 12]. In ADHD, ED is characterised by problems with temper control (feelings of irritability and frequent outburst of short duration) [13], emotional over-reactivity (diminished ability to handle typical life stresses, resulting in frequent feelings of being hassled and overwhelmed) [13], and mood lability (short and unpredictable shifts from normal mood to depression or mild excitement) [13].

According to the Diagnostic and Statistical Manual of Mental Disorders (DSM–5), the diagnosis of ADHD requires six out of nine ADHD symptoms of either inattention or hyperactivity/impulsivity in childhood, and five out of nine in adults (Table 1). Additional criteria include childhood age of onset defined as several ADHD symptoms present before the age of 12 years, pervasiveness defined as symptoms present in two or more settings, and impairment defined as- interference with or reduced quality of social, academic or occupational functioning [14].

**Table 1:** DSM-5 symptom criteria for attention deficit hyperactivity disorder [5]

Inattentive symptoms	Hyperactivity symptoms
• Fails to give close attention to details or makes careless mistakes in schoolwork, work, or during other activities • Has difficulty sustaining attention in tasks or play activities • Does not seem to listen when spoken to directly • Does not follow through on instructions and fails to finish schoolwork, chores, or duties in the workplace	• Fidgets with or taps hands or feet or squirms in seat • Leaves seat in situations when remaining seated is expected • Runs about or climbs, or is restless in situations where it is inappropriate • Unable to play or engage in leisure activities quietly • “On the go” acting as if “driven by a motor” • Talks excessively Impulsivity symptoms • Blurts out answers before questions have been completed • Has difficulty waiting turn • Interrupts or intrudes on others
• Has difficulty organising tasks and activities	
• Avoids, dislikes, or is reluctant to engage in tasks that require sustained mental effort • Often loses things necessary for tasks or activities • Easily distracted by extraneous stimuli • Forgetful in daily activities	
Associated features supporting the diagnosis	
- Emotional dysregulation (low frustration tolerance, emotional over-reactivity, or mood lability, as featured in the Wender-Utah adult ADHD criteria)^a^ - Mild delays in language, motor, or social development - Impaired academic or work performance^a^ - Increased risk of suicide attempts by early adulthood, primarily when comorbid with mood, conduct or substance use disorders^a^	

^a^Behavioural symptoms that commonly overlap with BPD diagnosis

The symptom profile and severity of ADHD varies greatly between individuals, with both inattention and hyperactivity/impulsivity associated with functional impairment in multiple domains [2, 15]. ED has also been found to be an independent predictor of impairment in ADHD, after controlling for the confounding effects of core ADHD symptoms (inattention and hyperactivity/impulsivity) on impairment [16–18]. Furthermore, this has been found in cases of ADHD with no co-existing mental health disorders, and therefore cannot be explained by co-occurring conditions [16]. Impairments can be severe, impacting on education, occupation, social and interpersonal relationships [2, 15]. Adults with ADHD are more likely to have lower educational attainment, poorer work performance and an increased likelihood of dismissal from work [19–21], as well as difficulties in maintaining long-term social relationships and higher divorce rates [22], serious transport accidents [23] and criminality [24].

ADHD seldom exists in isolation and up to 90% of adults with ADHD are reported to have one or more co-occurring mental health disorders [25]. Of these disorders, the most prevalent include mood, anxiety and substance use disorders [3, 26], and personality disorders including BPD [27, 28]. This exceptionally high co-morbidity rate could however reflect, at least in part, an artefact of overlapping symptoms shared by mental health disorders [12].

### Borderline personality disorder

BPD is a complex and severe mental health disorder, with typical symptom onset during adolescence and presence of behavioural precursors in childhood, persisting into adulthood [5]. BPD is characterised by a pervasive pattern of unstable interpersonal relationships, pronounced impulsive and self-damaging behaviour, unstable identity, and difficulties with ED [5], which substantially impact in an enduring way on quality of life and psychosocial functioning [29]. The DSM-5 diagnosis of BPD requires the pervasive presence of a minimum of five out of nine symptoms (Table 2) [5].

**Table 2:** DSM-5 symptom criteria for borderline personality disorder [5]

1. Frantic efforts to avoid real or imagined abandonment 2. A pattern of unstable^a^ and intense interpersonal relationships 3. Impulsivity in at least two areas that are potentially self-damaging^a^ 4. Identity disturbance: markedly and persistently unstable self-image or sense of self 5. Recurrent suicidal behaviour, gestures, or threats, or self-mutilating behaviour 6. Affective instability due to a marked reactivity of mood^a^ 7. Chronic feelings of emptiness 8. Inappropriate, intense anger or difficulty controlling anger^a^ 9. Transient, stress-related paranoid ideation or severe dissociative symptoms	
Associated features supporting the diagnosis	
• Recurrent job losses, interrupted education, and separation or divorce are common^a^	

^a^Behavioural symptoms that commonly overlap with ADHD diagnosis

In the general population, BPD has a prevalence of around 6% [30] and within populations of adult psychiatric inpatients, prevalence is around 20% [5]. Most epidemiological surveys report no gender differences of BPD, yet studies of clinical populations typically report higher prevalence figures in women than in men. The different sex ratios in clinical and population samples may be explained by both assessment and sampling biases [30].

Like ADHD, individuals with BPD commonly present with comorbid mental health disorders. In particular, around 90% of BPD cases are reported to have co-occurring mood disorders including depression and dysthymia [31], along with a high prevalence of substance use disorders in the range of 15% to 57% [32].

## Overlap in ADHD and BPD

### Studies of the co-morbidity between ADHD and BPD

Psychiatric comorbidity is commonly found across all mental health disorders [33] and is defined as the presence of two or more disorders in the same individual at a given time. In principle, each of the disorders should make a unique contribution to the clinical presentation of the individual [34]. However, estimates of comorbidity prevalence may be inflated if there is marked overlap in the symptom criteria of two disorders, leading to poor diagnostic delineation i.e. artefactual co-morbidity [35]. Furthermore, it remains unclear to what extent psychiatric diagnoses reflect entirely distinct disorders, rather than overlapping syndromes [34]. This is a particular problem for psychiatry since there are, as yet, no validated biomarkers or other objective markers with sufficient sensitivity or specificity to be used in clinical practice to distinguish aetiologically distinct mental health conditions. Regarding ADHD and BPD, while the specific symptoms used to classify the two disorders are different, many clinical characteristics are shared, including ED, impulsive risk-taking behaviour, and unstable interpersonal relationships.

A high prevalence of co-occurring ADHD and BPD is consistently reported in the literature. In a large in- and outpatient cohort of 372 adults with ADHD referred for ADHD assessment and treatment at a tertiary referral centre, 27.2% also met criteria for BPD assessed by the structured clinical Interview for DSM-IV II (SCID II) [36]. Similarly, in another sample of 335 adults referred by family physicians, community health clinics or self-referred, BPD, assessed by the SCID-II, was reportedly present in 10% of participants with DSM-IV inattentive subtype ADHD (six or more symptoms in inattention) and 24% of participants with combined subtype ADHD (six or more symptoms of both inattention and hyperactivity/impulsivity) [26]. Likewise, in a sample of 181 adult patients diagnosed with BPD by general practitioners and referred for treatment, 38.1% had comorbid ADHD, with 22.7% meeting the combined type criteria [37].

In a sample of 118 adult women from out-patient clinics seeking treatment for BPD, a high co-occurrence rate was reported: 41.5% met criteria for childhood ADHD (assessed retrospectively), and 16.1% met current criteria for the DSM-IV combined subtype, as well as meeting ADHD criteria as children [38]. However, as opposed to the previous studies where diagnoses was confirmed by clinical interviews [26, 36, 37], severity of borderline personality disorder and ADHD symptoms were assessed using self-report questionnaires [38].

In a sample of adolescents (*n* = 107) with emerging BPD drawn from a European research project investigating the phenomenology of BPD in adolescence, the prevalence of ADHD was 11%, an estimate that was not attenuated even when excluding symptoms of impulsivity accounting for possible symptom overlap [39]. This rate was close to the 16% rate found by Philipsen and colleagues, where current ADHD symptoms was assessed by self-report measures [38], as opposed to clinician-based interviews. Moreover, the samples significantly differed in regard to participants’ age.

Regarding population samples, results from the National Epidemiologic Survey on Alcohol and Related Conditions of more than *n* = 34,000 adults, found that lifetime comorbidity with BPD in the ADHD population was 33.7% compared with a lower prevalence of BPD of only 5.2% in the general population [40].

### Symptomatic overlap

There is considerable overlap in the symptoms of BPD and the associated features of ADHD (Table 3). Considering the onset and developmental trajectory, both disorders can be considered ‘developmental’ in the sense that they both emerge during childhood or adolescence and reflect enduring trait-like (non-episodic) symptoms and behaviours. The shared general features of trait-like symptoms that characterise both ADHD and BPD; means that differentiating between these diagnoses cannot easily be established by considering age of onset and course of symptoms. This means that to a large extent, differential diagnosis is based on the specific symptoms and behaviours used to define the two disorders.

**Table 3:** Overlapping features between ADHD and BPD

ADHD	BPD
• Childhood or early adolescent onset (note: recent literature highlights early adult onset in some cases) [115]	• Adolescent or early adult onset
• Chronic (trait-like) symptoms and persistent course	• Chronic (trait-like) symptoms and persistence course
• Pattern of unstable interpersonal relationships is a common associated characteristic	• Pattern of unstable interpersonal relationships
• Affective instability is common associated characteristic	• Affective instability
• Risk taking behaviour (behavioural impulsivity) is an associated characteristic	• Behavioural impulsivity/risk taking
• Inappropriate anger or difficulty controlling anger is a common associated characteristic	• Inappropriate anger or difficulty controlling anger

The most noticeable overlap among the core symptoms used to classify both conditions is impulsivity [1, 39]. Nevertheless, there are important qualitative differences in the manifestation of impulsivity used in the classification of ADHD and BPD. In ADHD, impulsivity refers to difficulty waiting or taking turn, blurting out during conversations (e.g. interrupting or talking over people), and intruding on others (e.g. butting into conversations or activities, taking over what others are doing) [5]. These impulsive symptoms are not always severe in adults with ADHD, but when severe can lead to impairment in social functioning and self-damaging or risk-taking behaviour. The consequences of severe impulsivity in ADHD include reckless driving, promiscuity, interpersonal relationship problems and aggressive behaviour [41, 42]. In BPD, impulsivity is defined by self-damaging behaviour, such as reckless driving, shoplifting, spending, binge eating, substance abuse and promiscuity [5]. People with either of these disorders may therefore display impulsive risk-taking behaviour, but from a diagnostic point of view they are core symptom of the BPD diagnosis, but only an associated feature of ADHD.

The other key area of symptom overlap is ED. This reflects a core symptom domain in the diagnostic classification of BPD [5], whereas in ADHD it is recognised as an associated clinical feature that supports the diagnosis [43, 44]. Nevertheless, ED is commonly seen to accompany ADHD, even in non-comorbid cases [35], and is an independent source of psychosocial impairment. This draws strong comparisons with ED in BPD, particularly when the ED that accompanies ADHD is severe [45]. At a descriptive level, the emotional symptoms of ADHD were well captured by Wender, Reimherr and colleagues in the earlier Wender-Utah criteria for ADHD, and show substantial overlap with the ED symptoms in the DSM-5 BPD criteria [1, 3, 38].

ED is a dimensional construct [46], referring to rapid and exaggerated changes in emotional states such as heightened irritability or hot temper [45]. A review by Asherson and colleagues reported that ED is present in 72–90% of adults with ADHD, and independently of other symptoms of ADHD predicts impairments in social, educational and occupational domains [47]. In contrast, ED is one of the core symptom domains of individuals with BPD, who nearly always suffer from severe persistent affective instability, inner tension and difficulty controlling emotions such as anger [27, 38, 48, 49]. Despite similarities, it has been suggested that patients with BPD have higher frequency and intensity of affective instability and aggressive impulsive reactions, compared to adults with ADHD [1, 49, 50]. Others describe ADHD patients as being high novelty seekers, who regulate their emotions through extreme external stimulation (e.g. sexual activity, aggressive behaviour), as opposed to those with BPD who tend to engage in self-mutilating behaviour to alleviate negative affect and inner tension [48]. However, self-harming behaviour and suicidality in ADHD has been highlighted in recent literature [51]. Yet, phenomenologically, ED is a complex construct, with shared characteristics in both ADHD and BPD, particularly pertaining to feelings of heightened anger and difficulty controlling anger (criterion eight in BPD) [38]. Others suggest that emotional instability reflects a similar cyclothymic temperament pattern in both disorders [52]. .Overall, it remains unclear whether the type of ED seen in ADHD really is qualitatively similar or different from that seen in BPD. One way to investigate this issue with precision is by using ambulatory assessments.

### ED in ambulatory assessments

Emotions are time- and context-dependent processes which are not adequately captured by retrospective and cross-sectional reports [53]. Yet, within clinical environments, assessment of ED relies entirely on interviews and self-report rating scales, which may be highly subjective and based on retrospective recall. These methods limit the validity of assessments of fluctuating emotional symptoms by the reliance on the individual’s memory, the skills of the interviewer, and may be coloured by their mental state at the time of the assessment [53, 54]. For instance, it has been reported that BPD patients fail to remember their most extreme and intense mood changes [55]. One approach with greater ecological validity is the use of ecological momentary assessments (EMA), also known as ambulatory assessments or experience sampling, which use repeated ratings of real time experiences [56]. EMA provides an effective way of precisely measuring emotional dynamics and variation within individuals, over time [57, 58].

In BPD, several EMA studies have investigated the dynamics of emotional instability [50, 53, 59–61]. In one study of 50 BPD and 50 healthy controls using 24-h ambulatory monitoring (intervals of 15 min), the BPD group was found to overestimate emotions with negative valence and underestimate emotions with positive valence, comparing retrospective with EMA ratings [60, 62]. In contrast, the healthy control sample overestimated emotions with positive valence and underestimated emotions with negative valence [60, 62]. Individuals with BPD have also been found to report greater levels of intra-individual variability and short-term fluctuations in overall affect valence. In another study comparing 34 outpatients with BPD and 26 with current depression, using EMA for nearly one month, ratings indicated greater instability (i.e. more changes from one assessment to the next) over time for fear, hostility and sadness in the BPD group [63]. It has also been reported using EMA that compared to healthy controls, BPD patients experience a higher frequency and increased intensity of negative affect and a lower frequency and decreased intensity of positive affect [50, 53, 60, 61]. In addition, a recent review of 34 EMA studies found that BPD patients experience longer duration of aversive tension and therefore a slower return to their baseline affective state [55].

To our knowledge, there has been only one EMA study looking at the dynamics of emotional instability in adults with ADHD [57]. Compared to healthy controls (*n* = 47), patients with ADHD (*n* = 41) showed significantly increased instability and intensity of negative emotions (irritability, frustration and anger). They also showed greater reactivity of negative emotions, such as anger, to ‘bad’ life events. This study included only males and specifically excluded patients with comorbid conditions [57].

Critically, from the standpoint of contrasting ED in populations of patients with ADHD and BPD, there have been no studies of the phenomenon in both patient groups using the EMA method. Furthermore, additional information could also be collected regarding the naturalistic context and situation when emotional changes occur (e.g. where they are, who they are with, what has just happened); which might identify disorder specific contextual triggers for emotional changes in different disorders. Clearly this area needs more research before conclusions can be drawn about the similarity or differences of ED in BPD and ADHD.

### Neurobiological correlates of ED in ADHD and BPD

The overlap in symptoms of emotional dysregulation in ADHD and BPD raises the question of a common neurobiological substrate for ED in the two conditions. In ADHD two competing hypotheses have been proposed for ED. First, the ‘dyscontrol hypothesis’ proposes that ED is driven by the same cognitive and neural processes that drive ADHD; for example, deficits in top down executive control, or bottom up state regulation factors [64]. In this model, ED reflects an alternative expression of the same underlying neurocognitive deficits that lead to ADHD symptoms. The alternative ‘affectivity hypothesis’ states that ED reflects deficits in neural processes related directly to emotional regulation, separate from those that lead to ADHD symptoms [64]. To date the accumulating evidence is pointing to the affectivity hypothesis. Two key publications support this conclusion [65, 66]. First, an investigation of cognitive performance deficits in ADHD (including inhibition, working memory, impulsive responding, slow and variable reaction times) found these were associated with ADHD symptoms independently from ED. [66] This suggests that different processes would explain the presence of ED in ADHD. Subsequently, a resting state functional Magnetic Resonance Imaging (fMRI) study in children with ADHD, found that ED, independently from ADHD, were associated with increased positive intrinsic functional connectivity (iFC) between bilateral amygdala and medial prefrontal regions, and reduced iFC between amygdala and bilateral insula/superior temporal gyrus. These findings suggested that ED is linked to disruptions in emotional control networks, which was not linked directly to ADHD [65].

Regarding BPD there are overlapping findings implicating the central role of emotional control networks. A critical review of fMRI studies conclude that emotional sensitivity, including emotional hypersensitivity and intense emotional reactions, was associated with increased amygdala activity and decreased activity with prefrontal cortical control regions [67]. In particular a consistent decrease in anterior cingulate activity and variable was identified, while the medial and dorsolateral prefrontal areas showed variable activity across studies. Overall, increased limbic and diminished prefrontal cortical activity suggested an impaired fronto-limbic inhibitory network [67].

Resting-state fMRI, contrasting intrinsic functional connectivity before and after an emotion regulation task in patients with BPD, further supports disrupted regulation of emotional circuits. Emotional hypersensitivity in BPD was associated with increased intrinsic connectivity between the amygdala and bilateral insula together with dorsal anterior cingulate cortex, while their impaired control over emotional reactions was associated with diminished intrinsic connectivity between the central executive fronto-parietal regions and salience network [68]. Overall the pattern of findings in relation to emotion regulation was similar to that reported for ADHD by Hulvershorn and colleagues [65].

The overlap of these findings in relation to ED in the two disorders suggests that there may be a common substrate for ED in the two conditions, involving altered top down and bottom up regulation of amygdala function and neural circuits. However, as we discuss below, evidence-based treatments are entirely different for the two disorders, suggesting that the underlying cause of the disrupted emotional circuits may differ in ADHD and BPD, potentially explaining differences in response to different treatments. Nevertheless, these findings suggest that there could also be common forms of treatment in a least a subset of patients with a comparable neurobiological basis for ED.

### Genetic and environmental risk factors

#### ADHD

It is firmly established that genetic factors play a central role in the aetiology of ADHD. The disorder aggregates among biological relatives of ADHD probands [69, 70], and twin studies estimate heritability in the range of 70–80% for parent and teacher ratings of ADHD symptoms in children, with similar estimates for clinically diagnosed cases of ADHD [69, 70]. In adults, self-rating of ADHD symptoms lead to lower heritability estimates in the range of 30–50% [71]. However, heritability estimates are similar to those seen in children for the clinical diagnosis of ADHD in adults, or when combining parent ratings and self-reports [71–73]. These studies find that the variance in ADHD in both childhood and adulthood is best explained by genetic and non-shared environmental factors, with no role for shared environmental factors independent of genetic influences [71].

Earlier candidate gene studies found significant associations with genetic variation within dopamine and serotonin system genes [74], although these have yet to be replicated using genome-wide approaches. Until recently genome-wide association studies (GWAS) of ADHD had not identified genetic variants that increase the risk of ADHD, although heritability due to the measured genetic variance was estimated to be around 30% [75, 76]. The most recent GWAS using a much larger sample of 20,183 ADHD cases and 35,191 controls identified twelve independent loci above genome-wide levels of significance (*p* < 5 × 10^− 8^), confirming the existence of numerous common variants of small effect that influence the development ADHD [77]. As these are recent findings, further research examining the role of these variants is required.

#### BPD

Though not as widely developed as the genetic literature on ADHD, there is a growing body of research implicating genetic influences in the aetiology of BPD. There is evidence to support familial aggregation of BPD features [78, 79] and findings from twin studies report heritability estimates in the range 35%–67% [80–82]. There is consensus between the studies that the remaining variance may be explained by unique rather than shared environmental influences, similar to ADHD.

To date there have been two GWAS studies of BPD. One study assessed two Dutch cohorts (*n* = 7125) using the Personality Assessment Inventory-Borderline Features Scale and found a promising signal on chromosome 5, which corresponds to SERINC5, a protein involved in myelination [83]. Seven single nucleotide polymorphisms (SNPs) in this region had *p* values between 3.28x10^− 6^ and 8.22x10^− 7^, while still remaining below genome-wide levels of significance [83]. The other more recent GWAS study was performed in *n* = 998 BPD patients and *n* = 1545 psychiatric controls [84]. While gene-based analysis yielded two significant genes for BPD, DPYD on chromosome 1 (1.20x10^− 6^) and PKP4 on chromosome 2 (8.24x10^− 7^), no genome-wide significant association was found for any SNP [84]. These specific findings in BPD do not overlap with findings from ADHD.

#### Common genetic risk factors for BPD and ADHD

Though there is evidence for symptom overlap between the two disorders, to date only one study has explored whether this could reflect overlapping genetic influences. Using a population twin sample, high phenotypic correlation (*r* = 0.59) was found between ADHD symptoms and borderline personality traits; consisting of four subscales- affective instability, identity problems, negative relationships and self-harm [85]. The authors found that the phenotypic correlation was explained by 49% genetic factors and 51% environmental factors, suggesting that shared aetiology could be a cause of comorbidity between ADHD and BPD traits [85]. However no further studies have been conducted looking at this relationship.

Overall twin studies of ADHD and BPD show a similar pattern of genetic versus environmental influences, with slightly higher heritability estimates in most ADHD studies. Yet it is important to note that heritability is also a functional of the reliability of the measures being used, with the residual non-shared environment including measurement error. Although for both ADHD and BPD there is no evidence for the main effect of shared environment (environmental effects shared by co-twins that explain co-twin similarity), shared environment may still play a major role through gene by environment interactions. It is therefore likely that for both disorders there are genetically driven individual differences in susceptibility to environmental stressors. The relatively high genetic correlation between ADHD and BPD is based on the correlation of trait scores in the general population, rather than diagnosed cases, but suggests a considerable degree of underlying shared aetiology that may explain the frequent co-occurrence of ADHD and BPD. Further studies are needed to investigate the genetic overlap between the two disorders, but also the overlap with specific symptom domains such as ED.

## Treatment approaches

Treatment approaches to ADHD and BPD are widely divergent. According to evidence-based clinical guidelines, in BPD there is limited evidence that medications reduce borderline personality symptoms, including ED, and psychological treatments are the cornerstone of treatment [86]. In contrast, in ADHD there is good evidence for effects of medication on reducing ADHD symptoms [87–89] and ED [90], and only limited evidence for effects of psychological treatments [91].

Clinical trials support the safety and efficacy of stimulants (methylphenidate, dexamphetamine,lisdexamfetamine) and atomoxetine, with reductions in the ADHD symptoms of inattention, impulsivity and hyperactivity, with moderate to large effects sizes ranging between 0.4 to 0.7 in adults [92–95]. In addition, several randomised controlled trials (RCTs) have evaluated the effects of pharmacological treatments on ED in ADHD patients, and found comparable treatment responses to the primary symptoms of the disorder [13, 17, 96]. These findings are further validated by the results of two recent meta-analyses which found moderate effects of stimulants (methylphenidate, dexmethylphenidate, amphetamines, lisdexamfetamine) and atomoxetine on ED in ADHD (average Cohen’s d across studies around 0.4) [90, 97]. In these studies, ED was assessed with various measures including ED subscales of the Wender Reimherr Adult Attention Deficit Disorder Scale, the Behaviour Rating Inventory of Executive Function, the Conner’s’ Adult ADHD Rating Scales and the Brown Attention Deficit Disorder Scale.

In contrast to treatment of ADHD, psychotherapy is regarded as first line treatment for people with BPD [5]. The most common therapies are Transference-focused Therapy [98], Schema Therapy [99], Mentalization-based Treatment [100], Systems Training for Emotional Predictability and Problem Solving, and Dialectical Behaviour Therapy (DBT) [101]. DBT, the most intensively studied intervention for BPD, significantly reduces anger (Standardized Mean Difference (SMD) = − 0.83) and self-harm (SMD = − 0.54), and improves overall mental health functioning (SMD = 0.65) [102]. Not only is psychotherapy regarded as first line treatment for BPD, UK NICE guidelines stipulate that pharmacological treatments should not be used for managing BPD, nor for individual symptoms or behaviours associated with the disorder [86]. The guidelines recommend the use of pharmacotherapy only as short term treatment measure during a crisis or in the instance of co-occurring mental health disorders [86].

Currently, there is insufficient data on the treatment of co-occurring BPD and ADHD. With regard to drug treatment, there have been no RCTs of stimulants or atomoxetine in BPD alone or in co-occurring BPD-ADHD cases [90].

There have however been only two case reports [103, 104] of successful methylphenidate treatment in patients with co-occurring BPD and ADHD, and two open-label studies [105, 106]; In one adolescent female-only study, patients with co-occurring ADHD and BPD (*n* = 14) reported significant improvement of BPD symptom severity (SMD = − 1.5) and aggressive impulsive behaviour (SMD = − 1.31) following treatment with methylphenidate for 12 weeks [105]. In a four-week study of 47 adults looking at effects of methylphenidate in addition to DBT, comorbid ADHD-BPD patients who were on stimulant medication (*n* = 24) showed a statistically significant improvement in anger control (SMD = 0.14), motor impulsiveness (SMD = − 0.62), depression (SMD = − 1.09) and ADHD severity (SMD = − 0.5), compared to those without medication (*n* = 23) [106].

Similarly, there are various psychotherapeutic treatments available for adults with ADHD, who are either unresponsive to stimulants and/or atomoxetine, or in need of adjunctive psychotherapy. There have been two exploratory open label studies [107, 108] examining effects of psychotherapy in adult ADHD. According to the multicentre open label study of *n* = 72 patients with ADHD, an adaption of DBT, addressing emotion regulation, depression, impulse control, stress management, neurobiology of ADHD and ADHD in relationships, DBT has therapeutic benefit for people with ADHD [108]. There was a statistically significant decrease on all psychometric measures in the study after DBT treatment; SMD = − 0.74 for the ADHD-Checklist, SMD = − 0.5 for the Beck Depression Inventory (BDI) and SMD = − 0.34 for the adapted Symptom Check List (SCL-16) measuring agitation, disorganised behaviour, emotion dysregulation and irritability among other traits [108]. Similarly, in the open label pilot study of *n* = 8 patients with ADHD, an adaption of cognitive behavioural therapy led to improvement in the same psychometric elements listed above; ES = 0.99 for the BDI, ES = 2.22 for the ADHD-Checklist and ES = 1.35 for the SCL-16 [107].

There have also been three RCTs of cognitive therapy [109–111] with relatively small sample sizes (*n* = 31, *n* = 43 and *n* = 51 respectively), addressing the effects of psychotherapy (in conjunction with medication in some cases) in adult ADHD that resulted in positive outcomes on all scales measuring severity of ADHD symptoms(ES = 1.2, d = 1.4 and, depression, anxiety, anger control and organisation skills among other outcomes. However, a recent large multicentre RCT of *n* = 433 adult ADHD randomised participants to group psychotherapy (GPT) developed and tailored to the treatment of ADHD, compared to clinical management (CM) reflecting optimal usual clinical care, with both groups randomised to methylphenidate or placebo [112]. While methylphenidate significantly reduced ADHD symptoms compared to placebo (*p* = 0.003), there were no significant differences in ADHD symptoms for those receiving GPT or CM (*p* = 0.16). In fact, in this trial, medication proved to be superior to intensive behavioural therapy, yet the latter resulted in better outcomes when combined with medication as compared with placebo [112].

Overall, while DBT modules and other systematically tailored psychotherapies appear to be helpful in ADHD, it is not yet clear whether they improve the core symptoms of ADHD (inattention, and hyperactivity/impulsivity), and there is insufficient data reported for effects on emotional dysregulation in ADHD [107–113]. This needs further investigation, since the evidence to date is based on relatively small studies, and there has been only one trial of cognitive behavioural therapy in ADHD samples without concomitant medication [112].

## Conclusions

In clinical practice, it should be acknowledged that the co-existence of ADHD with BPD may complicate the diagnostic process, and hinder treatment outcomes. Currently, patients with co-occurring ADHD and BPD are often seen by different specialists and provided treatments for one condition or the other, but only rarely for both. In fact, there is lack of empirical data to guide future clinical practice. Beyond the issues of differential diagnosis, there is insufficient awareness within specialist ADHD and BPD services of the potential benefits of treating the other condition. This needs to be addressed because treatment of both conditions may have positive benefits for individuals with overall better control of ADHD and BPD related symptoms and behaviours. Indeed, open clinical trials indicate the value of such a dual treatment approach.

Commonly in BPD patients with co-occurring ADHD, inattention and so called *executive function deficits* (i.e. sustained attention, forgetfulness, planning, organising, working memory), as well as physical restlessness and impatience, lead to difficulties in commitment and adherence to psychological therapies [114]. For example, this could be manifested in difficulties remaining seated, feeling restless and impatient, difficulties focusing on conversations and retaining information during therapy sessions, or insufficient planning and organisation to regularly attend therapy sessions [114].

A further potential benefit in a subpopulation of individuals with co-occurring ADHD and BPD may be a reduction in emotional dysregulation and impulsivity following medication treatment of ADHD. Similarly, psychotherapeutic interventions may be helpful for ADHD cases with high levels of emotional dysregulation with partial or no response to ADHD drug treatments, which could be accounted for by BPD. We therefore advocate a more nuanced approach to the management of people presenting with both ADHD and BPD.

An important question arising from the literature is the specificity of emotional symptoms that are seen in both ADHD and BPD. However, symptoms reflecting dysregulation emotional responses are also seen in other mental health disorders. A recent EMA study examined the dynamics of affective instability in patients with BPD compared with post-traumatic stress disorder and bulimia nervosa [56]. Using the same EMA protocol, all three conditions showed a similar degree of heightened affective instability regarding both the valence of emotional changes, and the level of associated distress [56]. Although BPD is the only disorder for which affective instability is part of the core diagnostic criteria [5], it seems that the specific dynamics of ED in BPD may not be so very different from that seen in other clinical groups.

Given the emerging genetic findings in relation to ADHD and BPD, and the overlap of symptoms such as ED, there may be gains from comparing the cognitive-neural underpinnings for ADHD and BPD, as well as overlapping symptom domains such as ED. At this stage, clinical trials are needed to evaluate the role of both ADHD medication and psychotherapy in the treatment of comorbid ADHD-BPD, and to identify treatment prognostic indicators. Under current circumstances, we suggest that health care professionals involved in diagnosing patients with either BPD or ADHD need to be aware of the potential diagnostic overlap and co-occurrence of these two disorders. Further, there should be sufficient clinical expertise to ensure that patient receive the evidence based treatments they require. This includes the potential benefits of drug treatments for ADHD, and psychotherapy for BPD.

## Abbreviations

ADHD: Attention-Deficit/Hyperactivity Disorder
BDI: Beck Depression Inventory
BPD: Borderline Personality Disorder
CM: Clinical Management
DBT: Dialectical behavioural therapy
DSM: Diagnostic and Statistical Manual of Mental Disorders
ED: Emotional dysregulation
EMA: Ecological momentary assessment
fMRI: Functional Magnetic Resonance Imaging
GPT: Group Psychotherapy
GWAS: Genome wide association studies
iFC: Intrinsic functional connectivity
RCT: Randomised controlled trial
SCID-II: Structured clinical Interview for DSM-IV II
SCL: Symptom Check List
SMD: Standardised Mean Difference
SNP: Single nucleotide polymorphisms
